# No evidence for self-recognition in a small passerine, the great tit (*Parus major*) judged from the mark/mirror test

**DOI:** 10.1007/s10071-017-1121-7

**Published:** 2017-07-31

**Authors:** Fanny-Linn Kraft, Tereza Forštová, A. Utku Urhan, Alice Exnerová, Anders Brodin

**Affiliations:** 1 Department of Biology, Lund University, Lund, Sweden; 2 Department of Zoology, Charles University, Prague, Czech Republic

**Keywords:** Mirror test, Self-recognition, Great tit, *Parus major*, Colour mark

## Abstract

Self-recognition is a trait presumed to be associated with high levels of cognition and something previously considered to be exclusive to humans and possibly apes. The most common test of self-recognition is the mark/mirror test of whether an animal can understand that it sees its own reflection in a mirror. The usual design is that an animal is marked with a colour spot somewhere on the body where the spot can only be seen by the animal by using a mirror. Very few species have passed this test, and among birds, only magpies have been affirmatively demonstrated to pass it. In this study, we tested great tits (*Parus major*), small passerines, that are known for their innovative foraging skills and good problem-solving abilities, in the mirror self-recognition test. We found no indication that they have any ability of this kind and believe that they are unlikely to be capable of this type of self-recognition.

## Introduction

Self-awareness, or the ability to recognize oneself as being separate from other individuals and the environment, is an advanced cognitive ability, previously considered as unique for humans and maybe some apes (Reader and Laland 2002; Dunbar and Schultz 2007). Many animals possess some degree of self-awareness in a cognitively trivial sense, for example knowledge of their own body parts when they navigate around obstacles (Shettleworth 1998). Also, self-recognition in a mirror may not be an all-or-nothing ability as some animals seem to have an understanding of mirror reflections even if they do not pass tests of mirror self-recognition (e.g. Pepperberg et al. 1995; Clary and Kelly 2016). Here, we will only consider self-awareness in the form of an advanced cognitive trait that according to some authors would require possession of a theory of mind (Gallup 1998; Shettleworth 1998; but see Suddendorf and Butler 2013).

The most common way of investigating whether an animal is self-aware is to test whether it is being capable of mirror self-recognition (MSR). This is usually performed with the mark/mirror test (Gallup 1970). This test involves application of a mark on the body of an animal in a place that is not visible to the animal without a mirror, such as the face or chest depending on the species tested (Gallup 1970; Plotnik et al. 2006; Prior et al. 2008). Only humans that have reached a certain age understand mirrors directly (Amsterdam 1972; Broesch et al. 2011), so before the test the animal must first become accustomed to seeing itself in a mirror (Gallup 1970; Plotnik et al. 2006; Uchino and Watanabe 2014). Even species that we consider to be cognitively advanced such as the chimpanzee (*Pan troglodytes*) may behave as if the reflection is another individual up to 5 days after the introduction of a mirror (Gallup 1998). After mirror exposure, the mark is placed on the animal in a controlled way, for example under anaesthesia, and the animal is subsequently allowed to observe itself in the mirror. A positive response will then be an increase in self-directed (or specifically mark-directed) behaviour, for example attempts to remove or scratch the mark (Gallup 1970; Plotnik et al. 2006).

Individuals of several mammal species have been demonstrated to be capable of MSR, such as chimpanzees (Gallup 1970), Asian elephants (*Elephas maximus*) (Plotnik et al. 2006) and bottlenose dolphins (*Tursiops truncatus*) (Reiss and Marino 2001). In birds, the ability has been demonstrated only in the Eurasian magpie (*Pica pica*) (Prior et al. 2008), whereas other cognitively advanced species such as the African grey parrot (*Psittacus erithacus*) and the jackdaw (*Corvus monedula*) have failed the mark test (Pepperberg et al. 1995; Soler et al. 2014). For a recent review of self-perception in birds, see Derégnaucort and Bovet (2016). Recently, rhesus macaques (*Macaca mulatta*) (Chang et al. 2015) and pigeons (*Columba livia domest.*) (Uchino and Watanabe 2014) have passed the MSR test after extensive training (but see Ünver et al. 2017). These are species that previously have not passed the test, and it is questionable whether this is self-awareness according to Gallup (1970, 1998) since both studies appear to involve conditioning to the mark rather than comprehension of the situation in a cognitive sense. It has been demonstrated previously that conditioning will make it possible for pigeons to pass the MSR test (Epstein et al. 1981). Clark’s nutcrackers (*Nucifraga columbiana*) did not pass the test in the typical set-up but behaved as if they understood their own reflection in a blurry mirror (Clary and Kelly 2016).

In most species that show evidence of MSR, only some individuals will pass the test (Gallup 1970; Plotnik et al. 2006; Prior et al. 2008), whereas others seem to be unable to learn that it is themselves they see in the mirror. Alternatively, it is possible that all individuals in a population possess similar abilities of self-recognition but that the typical set-up of the mark/mirror test is not suitable for all individuals. Regardless of what the reason is, the result may be a strong effect in a few individuals rather than a smaller and more even effect across a large group (Gallup 1970; Plotnik et al. 2006; Prior et al. 2008).

The great tit (*Parus major*) is a small passerine of the *Paridae* family found all over Europe as well as parts of Asia and Africa (Cramp and Perrins 1993). There has been no previous study of MSR in parids, even if aggressive reactions towards mirrors have been studied in other species in this family (Censky and Ficken 1982; Branch et al. 2015). Great tits are considered to be innovative and opportunistic foragers that, for example, have been recorded to use tools to extract food from bark crevices (Lefebvre and Boogert 2010; Cauchard et al. 2013; Brodin and Urhan 2015). They are better learners and problem solvers than other species in the family (Sasvári 1979), and they are unusually good observational learners (Brodin and Urhan 2014; Aplin et al. 2015). Also, on a more general level, parids have relatively large brains compared to most other passerine families (Lefebvre and Boogert 2010) and high numbers of neurons in the forebrain (Olkowicz et al. 2016). Taken together, these traits indicate a high cognitive ability, and if any small passerine should be expected to pass the mark/mirror test, we consider the great tit a likely candidate.

## Materials and methods

We conducted this study from the beginning of October 2014 to the end of March 2016 at the Department of Biology, Lund University, Sweden, and at the Faculty of Science, Charles University, Prague, Czech Republic. The study was started as two separate studies: one in Lund and one in Prague. Half-way through these, we realized that we were doing the same test on the same species, great tits, with methods taken from the same paper (Prior et al. 2008). As the results were very similar, we decided to join the studies and pool the results even though there were some minor differences in the methods. As far as we can see, none of these differences affected the outcome of the mark/mirror test in any way. It had some effects, however, in how the birds responded to training. The main differences were that: (1) in Lund all birds were captured in the wild, whereas in Prague half of the birds were captured in the wild, but the other half was raised in the laboratory, (2) in Lund we used paint marks but in Prague we used paper stickers, and (3) in Lund we used one colour, bright yellow, in a 20-min test session, whereas in Prague we used two colours, pink and bright blue, in two consecutive 10-min sessions.

### Test subjects

#### Wild-caught birds

In Lund, we tested 18 individuals, of which ten were male and eight were female. Nine of the birds were young (first autumn or winter) birds and nine were adults. In Prague, we tested 20 birds, of which ten were males and ten were females. Twelve of these were adults and eight were young birds. We captured birds during the non-breeding season (September–March) near Lund (55°42′N, 13°11.8′E) in Scania, the southernmost region of Sweden, and in Prague (50°50′N, 14°25′) in Central Bohemia, Czech Republic. During autumn and winter, great tits form foraging flocks, in which social interactions are frequent, and the birds are therefore motivated to explore the mirror images perceived, at least at the first sight, as another individuals. We attracted the birds either by playback recordings of great tit song, or by feeders, and then captured them with mist nets. We then transported them to the laboratories in individual cotton bags within 30 min after capture. At capture, we equipped the birds with plastic colour rings for individual identification. During captivity, we kept the birds in individual cages in special bird facilities (see below). After the experiment, we removed the colour bands and released the birds at the original place of capture. We allowed the birds to adjust themselves to their cages and the laboratory environment for 2–3 days before we started training sessions. As we did all training and experimental sessions on separate days, this resulted in the birds being kept in the laboratory for a maximum of 2 weeks before they were released.

#### Hand-reared juveniles

Aside from the wild-caught birds we hand-reared 20 birds in Prague. We took these birds from nest boxes situated in large parks at Prague’s outskirts. The nestlings were taken when 12–14 days old (one or two birds per brood). In the laboratory, we kept them in artificial nests and fed them every 2 h from 6 AM to 10 PM for several days, until they were able to feed themselves. Their diet consisted of mealworms (*Tenebrio molitor* larvae), hand-rearing food mixture (Orlux Handmix), egg mixture Oké-Bird (Versele-Laga) and other mixtures for insectivorous birds with vitamins and minerals added. After fledging, we housed these birds in the same type of home cages as the adult birds, first in groups of 3–4 birds and later on individually. All birds were tested when we considered them to be fully independent, at the age of 46–65 days. After the experiment, we released them in the locality of their origin. While wild-caught birds could have encountered reflections of themselves in water surfaces or car mirrors, we see no risk of this in the hand-raised birds.

### The facility

We used specially designed bird rooms in the animal facilities at the biology department of Lund University and at the zoology department of Charles University for all sessions. We kept the wild-caught birds at a room temperature of 14–18 °C and under a light regime of 10 L:14D. We kept the hand-reared birds at a temperature of 20–24 °C and under a light regime of 16L:8D. The lights had a daylight spectrum with a 1-h dimming function at dawn and dusk. The bird cages were 40 cm wide × 60 cm long × 60 cm high in Lund and 40 cm wide × 50 cm long × 50 cm high in Prague. The birds had ad libitum access to a birdseed mixture and netted suet cakes supplemented with mealworms and commercial food for insectivorous birds as well as water enriched with a commercial vitamin mix for birds. We cleaned the cages every day. During experimental sessions, we observed the birds from an observation booth behind a dark-tinted window that allowed observations of the birds without disturbing them.

### Training and testing

**Fig. 1:**
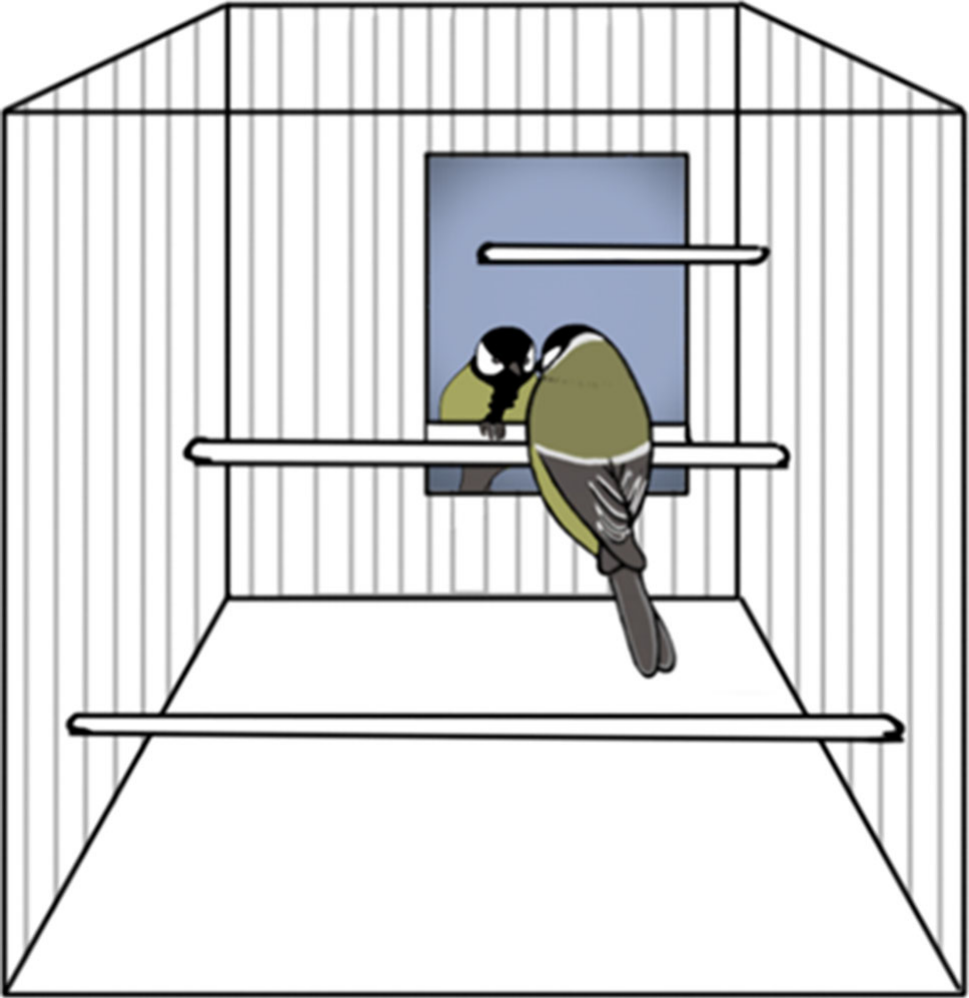
Position of the mirror in a great tit’s home cage. Observe that the drawing is not made to scale; the mirror was smaller than it appears in the figure (see text)

The study consisted of two phases: five training sessions and then a mark-test session. We recorded all training and test sessions in Lund with a Sony Action Cam HDR-AS200VT and in Prague with a Canon HG20 camera. The cameras were mounted approximately 20 cm from the cage’s short end angled slightly downward so that they provided good views over the whole cages and their floors. Training sessions lasted 20 min in both Lund and Prague, and we started each such session by introducing a vertically positioned mirror in the focal bird’s home cage (Fig. 1). The mirrors we used were 15–18 cm high and 10–16 cm wide and mounted against a wall of the cage. As it was hard to determine precisely where a bird was looking or sometimes even its specific behaviour, we analysed the bird’s behaviour afterwards by watching the recordings. The playback was repeated or slowed down if the behaviour of a bird was difficult to categorize. We categorized the bird’s behaviour in accordance with a similar study on birds (Prior et al. 2008) (Table 1). Between the sessions, we removed the mirror from the cage to minimize the risk of uncontrolled effects. In accordance with Prior et al. (2008) and Soler et al. (2014), we started to analyse both the frequencies of the various behaviours and the times spent on them. Early in this process, we realized that the frequencies became very misleading. Great tits move very quickly in their cages with behavioural elements changing dynamically. Typically a bird that appeared not to be interested in the mirror could move rapidly around in the cage perching in front of the mirror every time it passed it, getting a high count. A bird that appeared to be interested in the mirror would typically perch for an extended time in front of the mirror, getting a low count. Furthermore, we believe that time scores are more robust when it comes to inter-observer reliability in agile little birds like great tits. This is important as we have merged data from two laboratories. It is possible that the wild-caught birds could have experienced mirror reflections of themselves outside the laboratory, for example in water surfaces or car mirrors. This, however, would not be a problem since it could only make them more accustomed to mirrors.

**Table 1:** Various behaviours we recorded from the video analyses

Category	Behaviour	Description
Mirror interest	Looking at mirror	Spending time on perch in front of mirror or next to mirror while looking into mirror
Social behaviour	Submissive display	Crouching and turning black frontal markings away from mirror
	Aggressive display	Displaying black frontal markings to mirror
	Attack	Flying into mirror
Body-directed behaviour	Preening	Using the bill to preen feathers outside the mark area
	Cleaning bill	Wiping the bill against the perch
	Ruffling	Shaking the feathers to separate and fluff them
	Scratching	Scratching any part of the body except for the mark with a foot
	Body grooming	
Mark-directed behaviour	Mark grooming	Using the bill to preen feathers at the mark
	Mark scratching	Scratching the mark area with a foot

**Fig. 2:**
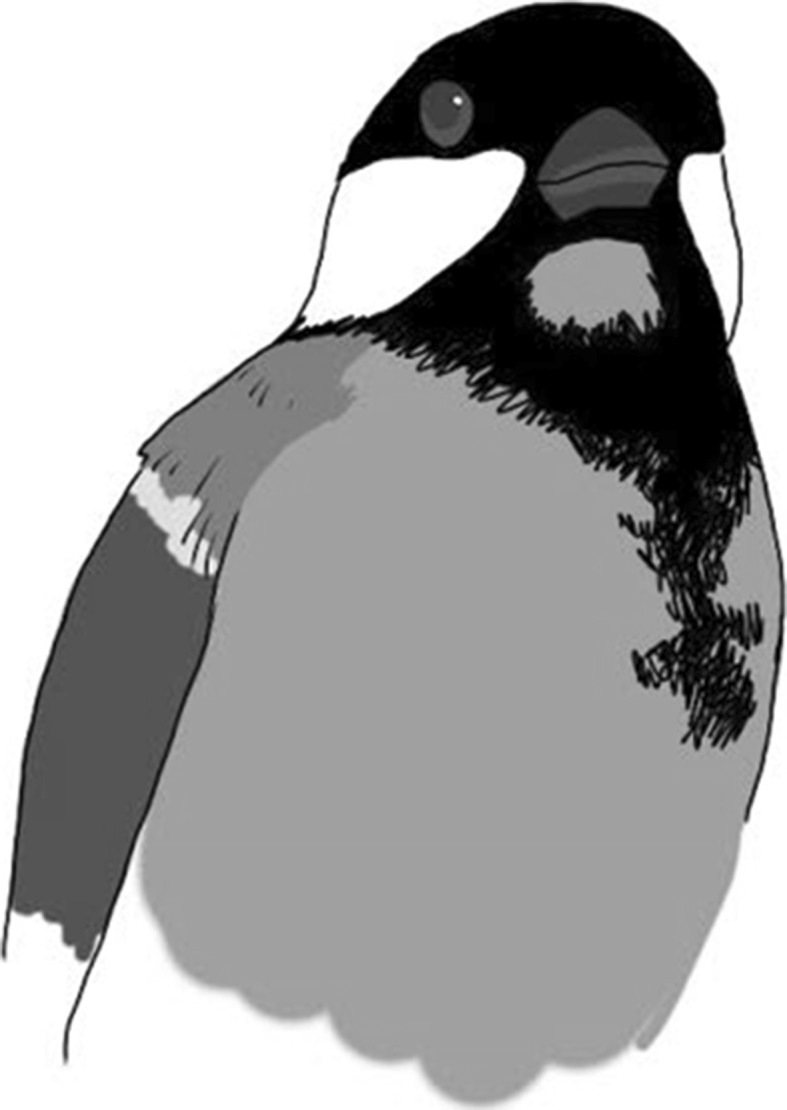
Position of the *yellow* mark on a great tit’s chest. The *blue* and *pink* marks and *black* sham mark were positioned in the same way (colour figure online)

Upon completion of training, we marked the bird with either a coloured or black mark on the uppermost part of its chest (Fig. 2) where it cannot be seen directly by the bird. During the colour application process, one experimenter held the bird in one hand and applied the mark with the other. We held the bird with its breast upwards and the back of the hand resting against a desk. Holding a small bird this way makes it easy to cautiously fixate its head by holding the index finger and the thumb on each side of the beak. This procedure means that the fingers of the experimenter will block the bird’s view of the underparts of its body so that it cannot see the application of the mark. We used this mark location in accordance with Prior et al.’s (2008) experiment on magpies. The black mark was a “sham” mark as control against tactile effects since the area around the position of the mark is black on the great tit. We tested several types of dye and tapes before the experiments in order to find some that appeared not to be sensed by the birds. We evaluated this by releasing the marked bird in its home cage and observed whether it scratched or preened the spot with the mark. In Lund, we ended up with a dye that was an eyeliner of the brand H & M (Hennes & Mauritz). In Prague, we used small equilateral triangles (with edges 4 mm) cut of a thin paper masking tape, which stuck lightly to the feathers. As none of these markings appeared to be sensed by the birds, we do not think that this difference affected the results. We did not include the birds used in the initial testing of dyes and tapes in the main study.

The procedure in the mark-test sessions differed slightly between Lund and Prague. In Lund, we carried out a single 20-min session with a conspicuous yellow-coloured mark. In Prague, we tested two different conspicuous colours, pink and bright blue, each in a 10-min session. After each such colour session followed, in both laboratories, a session with the black “sham” mark. In Prague, there were therefore two sessions with the sham mark, corresponding to the two colour-mark sessions. Before we started a mark-test session, we removed the food from the cage as food is a possible source of distraction. We then captured the bird in the cage, marked it, put it back again and allowed it to rest for 1 h. We then filmed each bird for 10 (Prague) or 20 (Lund) min before we introduced the mirror and, subsequently, for 20 min (split up in two 10-min sessions in Prague) with the mirror introduced. A similar procedure has been used in previous mirror mark tests in birds (Prior et al. 2008; Uchino and Watanabe 2014).

### Analyses

Using the video recording of the tests, we counted all self- and mirror-directed behaviours within each session in accordance with Table 1. We noted the time spent performing each behaviour in seconds and measured all behaviours as whole second events. If a bird appeared to behave as if its mirror reflection was another individual, we categorized this as social behaviour (e.g. Prior et al. 2008). If a bird appeared to show mirror interest and social behaviour simultaneously, we categorized this as social behaviour. We did not include singing and other vocalizations in the analyses since it was very hard to determine whether the bird was directing such signals towards the mirror. We categorized self-directed behaviours, such as preening, as either being directed towards the mark or to the rest of the body.

**Fig. 3:**
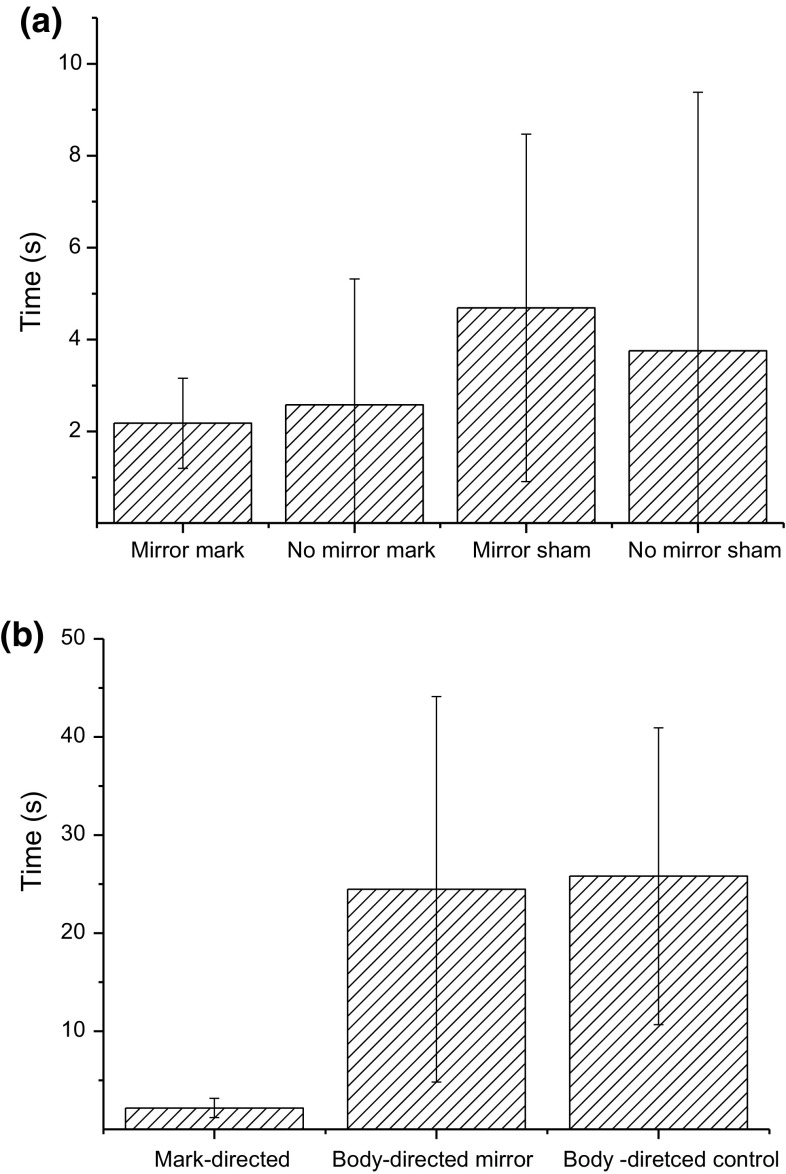
Time spent on preening and scratching the mark in the mirror test compared to **a** the control and sham-mark sessions and **b** compared to the time spent on preening and scratching other parts of the body (*n* = 58). Observe that the first *bar* shows the same data in **a** and **b**. The dispersion measure is 95% confidence intervals. The difference between *columns 1* and *2* in *b* is tested in Table 2

We divided the behaviour during training and experimental sessions in discrete categories according to Table 1. Since the variance tended to increase with the mean length of the time period (e.g. Fig. 3), we log-transformed data before statistical analyses. Also, as the data contained zeros (e.g. if a bird perched with no recordable behaviour), we added one second to each observation (log *x* + 1), since log 0 is undefined (Hampton 1994). To test for an effect of training on mirror interest and social behaviour, we used repeated-measures ANOVA with the intention of continuing with a linear regression as a follow-up test in case of significance. It was hard to develop a balanced repeated-measures ANOVA with all treatments and factors as the factor that proved to be the most important (rearing condition) only occurred in Prague. Therefore, we had to make several *t* tests and ANOVAs for the analyses. We used 95% confidence intervals as dispersion measure, both in the text and in the figures. With the colour marks and the black sham mark, we had the following four data categories: colour mark/mirror, colour mark/no mirror, sham mark/mirror and sham mark/no mirror, which we compared in paired *t* tests. The crucial “self-recognition” test will then be between colour mark/mirror and colour mark/no mirror, whereas the sham mark will serve as a control for tactile effects of the mark. We also tested for effects of some other possible confounding factors such as time of year, but as none of these factors tended to approach significance we do not bring them up in the discussion. All tests are two-tailed with the threshold for significance set at *p* ≤ 0.05.

As the results occur in the form of two 10-min sessions in Prague rather than as one 20-min session, we combined the data from the two 10-min sessions. This sum can then be seen as a response to colour (bright blue and pink) rather than as two responses to two different colours. The birds’ reactions to pink and bright blue were very similar. The rationale for this treatment is twofold: (1) the birds in Prague would otherwise be included twice in the tests, and (2) the session duration will be the same as in Lund.

## Results

### Training sessions

There were two measures that could indicate the birds’ interest in the mirror: mirror interest and social behaviour towards the mirror. It may be expected that these measures initially would be high and then decrease over sessions if the birds got habituated to mirrors. There was no such trend in either mirror interest (RMANOVA, *F*_4,57_ = 0.816, *p* = 0.52) or social behaviour (RMANOVA *F*_4,57_ = 1.474, *p* = 0.21). The only significant effect we could find was that the hand-raised birds spent significantly longer time (265.9 ± 60.7 s) looking in the mirror than the wild-caught birds (135.7 ± 38.1 s, *two*-*sample t test*, *t*(19) = 2.78, *p* = 0.007). This could depend on either the hand-raised birds being young (i.e. an age effect) or being more accustomed to their cages than the adult birds and therefore more interested in novel stimuli including mirrors (i.e. an effect of rearing conditions). It appears to be an effect of rearing conditions, rather than age, as the wild-caught young birds did not look longer in the mirror (131.6 ± 49.1 s) than the adult birds (139 ± 38.1 s, *two*-*sample t test*, *t*(16) = 0.11, *p* = 0.91).

### Mark test

The birds did not spend more time on colour-mark-directed behaviours (preening or scratching the mark) when the mirror was present (2.18 ± 0.98 s) compared to when no mirror was present (2.58 ± 2.74, Fig. 3). Even though the mean was slightly higher in the control sessions than in the test sessions, we made a paired *t* test for a difference (*paired t test*, *t*(57) = 0.35, *p* = 0.725). Neither was there any effect if we separated data between Lund and Prague. In Lund, the birds spent 3.56 ± 2.42 s on mark-directed behaviour with the mirror present and 5.0 ± 4.04 s when it was not present. In Prague, they spent 1.56 ± 0.88 s on mark-directed behaviours with the mirror present and 1.50 ± 1.72 s without it. There was a non-significant tendency for a difference between Lund and Prague (*two*-*sample t test*, *t*(17) = 1.9, *p* = 0.062) with the mirror present. As it is possible that pooling of the data could increase noise and hide effects, we also made a repeated-measures test with city as a factor to control for this, but there was no effect of city (RMANOVA *F*_2,57_ = 1.48, *p* = 0.29). The hand-raised birds spent 2.65 ± 1.06 s on mark-directed behaviours and the wild-caught spent 1.95 ± 1.26, a non-significant difference (*two*-*sample t test t*(17) = 0.64, *p* = 0.52).

We also compared the times spent on mark-directed behaviour in mirror and control sessions for the birds individually. Three birds in Lund spent notably longer times than other birds on such behaviour in the mirror sessions, 15, 14 and 10 s, respectively. These birds, however, spent correspondingly longer times on body-directed behaviours (61, 42 and 45 s, respectively), suggesting that this was a general increase in preening/scratching and not a mirror effect. Also, these birds did not perform these behaviours in a way that suggests that they used the mirror for it, for example scratching or preening when watching (or in close conjunction to watching) themselves in the mirror. It should be noted that without these birds, the mean in Lund was 1.66 ± 1.44, which was lower than the mean in Prague.

We intended the sham mark to be a control for an increase in mark-directed behaviours depending on tactile effects of the mark rather than the birds seeing it in the mirror. As we found no mirror effect, we do not need such as control but we still include it here for comparison. When the birds had the black sham mark, they spent 4.69 ± 3.78 s on mark-directed behaviours in the sessions with a mirror; this was not significantly different from the 2.18 ± 0.98 s they spent on these behaviours when they had the colour mark (*paired t test*, *t*(57) = 0.34, *p* = 0.73). When the birds were carrying the sham mark, they did not direct more behaviour towards the mark with the mirror present (4.69 ± 3.78 s) compared to when it was not present (3.76 ± 5.62 s, *paired t test*, *t*(57) = 0.41, *p* = 0.68).

Self-recognition in a mirror could manifest itself not only as a difference between the mirror/no mirror sessions, but possibly also as an increase in colour-mark-directed behaviour relative to similar behaviours directed to other parts of the body. In these comparisons, the results were significant, but in the opposite direction of the prediction, as the birds preened and scratched themselves more on the rest of the body and then on the colour-mark region (Table 2). This should be expected, however, if there is no self-recognition effect as “the rest of the body” is a much larger area than the mark region. It is possible that the proportion time spent on mark-directed behaviour out of the total time spent on preening/grooming, etc. could be more sensitive to changes between experimental conditions. This proportion was 0.07 ± 0.03 with the mirror present and 0.05 ± 0.02 in the control sessions, a non-significant difference (*paired t test*, *t*(57) = 1.16, *p* = 0.25). None of the factors sex, age, rearing condition (hand-raised/wild-caught) or local place of capture showed any tendencies for any effect in this respect or in most other tested effects (not shown). The only effect we could find was, just as during training, that the hand-raised birds differed from the other birds in some respects, for example they spent more time looking in the mirror (430.9 ± 97.3 s) than the wild-caught birds from the same population (190.5 ± 116.9 s, *two-sample t test*, *t*(16) = 5.0, *p* < 0.001). This, however, appeared to have no relationship to self-recognition as the time they spent looking in the mirror was not significantly different (425.2 ± 89.5 s) than with the sham mark (*paired t test*, *t*(19) = 0.225, *p* = 0.824).

**Table 2:** Time spent on preening and scratching the chest (the colour-mark area) compared to preening and scratching the rest of the body

Birds	Time mark	Time body	*n*	*t*	*p*
Pooled	2.18 ± 0.98	24.48 ± 19.65	58	13.81	<0.001
Lund	3.46 ± 2.42	19.89 ± 10.17	18	10.67	<0.001
Prague	1.56 ± 0.88	26.55 ± 22.72	40	11.39	<0.001

Out of the 58 birds tested, one individual (a juvenile bird from Prague) removed the mark during the experiment. In this case, it was the sticker mark, and it was removed at the very end of the mirror session, so the mark was present for the most of time. In all other cases, the marks remained at place for the whole time of testing.

## Discussion

None of our results indicate that great tits have any ability to recognize themselves in a mirror. If the birds had been capable of MSR, we would have seen an increase in mark-directed behaviour in the “mark/mirror” condition, but there were no such tendencies whatsoever. All experimenters were aware that MSR ability in most species will only occur in some individuals, and eager to find evidence for this ability, we checked carefully for this, but we found no such tendency in any video recording.

If great tits are capable of MSR, but we have failed to detect this, we can see two reasons for this. Either the effect could be so small that we could not detect it, or somewhat more likely, the ability occurs only in some individuals. Both of these alternatives are less likely for several reasons. First, there is no evidence from other studies that MSR ever should occur as a weak, general effect that is hard to detect (Plotnik et al. 2006; Prior et al. 2008; Chang et al. 2015). Instead, MSR is usually expressed strongly in some individuals, whereas others show no evidence of it (Plotnik et al. 2006; Prior et al. 2008; Chang et al. 2015). Our sample size of 58 birds is considerably larger than in most other studies of MSR in which usually only a few individuals have been tested. Based on previous studies, Clary and Kelly (2016) estimated that ten individuals should give sufficient power to detect a cognitive ability of this type. In most previous studies, the effect has been very strong in the individuals that have displayed MSR. Hence, we consider it likely that we would have encountered at least some individuals capable of MSR with our (in this context) large sample size (Gallup 1970; Plotnik et al. 2006; Prior et al. 2008). That we should have missed a weak effect seems even more unlikely as there were not even any tendencies for the means to go in the right direction (Fig. 3).

Considering that the great tit has shown abilities suggesting it to be more cognitively advanced than one could expect (Sasvari 1979; Brodin and Urhan 2014; Aplin et al. 2015), we were hoping that it would pass the MSR test when we started this study. A closer look on previous studies, however, tells us how hard this test actually is for animals. According to de Waal (2008), hundreds of species have been tested for MSR, but only a handful has shown any evidence for it. In the studies that have succeeded in demonstrating MSR, usually only a few individuals have passed the test. Gallup (1970) did not present individual data on the four chimpanzees he tested, but in a later, larger study, 10 chimpanzees out of 30 passed the test (Povinelli et al. 1993). Out of five tested Indian elephants, one passed the test (Povinelli 1989; Plotnik et al. 2006). Two bottlenose dolphins that were tested both passed the test, but these had been living in a mirror-coated pool for years before the test (Reiss and Marino 2001). Five of seven rhesus monkeys showed mirror-induced self-directed behaviour, but these had been conditioned to “spot-touching” with extensive training before the test (Chang et al. 2015). In the only bird species in which MSR has been demonstrated, the magpie, two (or possibly three) out of five tested individuals showed evidence for MSR (Prior et al. 2008). Given that an effect was detected with such small sample sizes, one would expect that at least a few of our 58 great tits would have passed the test if our great tits possessed this ability. Not only was our sample size unusually large but we tested birds from two distant populations, males and females, young and adult birds, hand-reared and wild-caught birds and birds with sticker marks versus birds with paint marks. Also, we used three different colours in the tests. We therefore believe that our results are general and representative for the great tit.

We still think that the great tit was a logic choice as a candidate to pass the mark test as it is known to be more innovative and better at learning than its relatives in the Paridae (e.g. Sasvári 1979). Since we found no evidence for MSR in a species as the great tit, we think that it is less likely it should occur in other similar passerine species. It is still possible that the great tit possesses some ability for MSR but that the all-or-nothing nature of a pass-and-fail test of MSR is not suitable to detect this. For example, individuals of some species might be less motivated to remove marks than those in others. Since animals seldom will see clear and detailed reflections of themselves in nature, Clary and Kelly (2016) allowed Clark’s nutcrackers to cache nuts in front of blurry and clear mirrors. The nutcrackers behaved as if they realized that the blurry reflections were themselves even though they did not react to colour spots on themselves in the clear mirrors. This suggests that animals may have some understanding of their own mirror reflections even if they cannot understand how to preen themselves with guidance of a mirror.

It is of course of overriding importance in a study of MSR that the marks are not sensed tactilely by the birds. Soler et al. (2014) discussed the problem with confounding tactile effects by stickers and suggested that dyes might be better as markers. We used both stickers (Prague) and dyes (Lund), and the amount of mark-directed behaviours tended to be higher in Lund. In contrast to the studies of Prior et al. (2008) and Soler et al. (2014), none of our markers appeared to be sensed by the birds, neither when we tested them before the experiment, or in the experiment. It is possible, however, that applications of dye will differ more between individuals than stickers and that some of the dye applications may be more prone to get sensed by the birds.

Besides technical issues with the marking, there may also be problems with the interpretation of animals’ behaviour during the MSR test. Even in a species such as the chimpanzee, it may be difficult to decide whether a specific behaviour is directed towards the mark and how various behaviours should be interpreted (e.g. Heyes 1994; Povinelli et al. 1997). Also, it may be important for animals to have the opportunity to look behind the mirror in order to understand its function. As the mirror was attached to the external wall of the cage, this was not possible for the birds in our study. Still we think that the risk that we have missed MSR ability is very small in our study.

In conclusion, we found no evidence that the great tit should be able to understand its own mirror reflection. This suggests that it is incapable of MSR, at least in the typical mirror test set-up. This does not rule out, of course, that the great tit possesses some kind of self-awareness that is not detectable in the mirror test.
